# Assessment of allopurinol use in patients with chronic kidney disease and asymptomatic hyperuricemia: a retrospective cohort study

**DOI:** 10.1590/2175-8239-JBN-2025-0243en

**Published:** 2026-04-20

**Authors:** Maria Elaine Latosinski Santos de Souza, Tiago Antônio Heringer, Ana Paula Helfer Schneider, Lia Gonçalves Possuelo, Andreia Rosane de Moura Valim

**Affiliations:** 1 Universidade de Santa Cruz do Sul, Santa Cruz do Sul, RS, Brazil.

**Keywords:** Uric Acid, Allopurinol, Hyperuricemia, Renal Insufficiency, Chronic

## Abstract

**Introduction:**

Asymptomatic hyperuricemia (AH) is common in patients with chronic kidney disease (CKD) and has been identified as a modifiable condition. Addressing it could increase the possibilities for preventing and treating kidney injury.

**Objective:**

To assess whether administering allopurinol to people with chronic kidney disease and asymptomatic hyperuricemia enhances kidney function and delays progression to kidney failure.

**Methods:**

This is a retrospective cohort study with data collection from medical records at a specialized center from 2006 to 2020. Eighty people in stages 3 and 4 of CKD with AH were divided into two groups: 40 patients received allopurinol and 40 did not receive medication. Patients were followed for 24 months, with four consultations. Serum uric acid levels and creatinine-based estimated glomerular filtration rate (eGFRcr), estimated by the CKD-EPI formula, were compared within groups and between groups using mean, standard deviation, and analysis of variance (ANOVA).

**Results:**

Eighty patients were included in the study. Comparing 40 patients with no treatment for hypertension with 40 patients receiving hypouricemic therapy (HUT) with allopurinol, it was observed that mean serum uric acid decreased at all review visits in the allopurinol group, and there was a significant increase in mean eGFRcr after starting allopurinol (p < 0.001). None of the patients in the allopurinol group developed end-stage kidney disease (ESKD) within 24 months (p < 0.001). Similar results were not observed in the control group.

**Conclusion:**

Allopurinol was useful in reducing serum uric acid levels, improving kidney function, and delaying progression to kidney failure in patients with AH.

## Introduction

Patients diagnosed with chronic kidney disease (CKD) and managed on an outpatient basis frequently exhibit asymptomatic hyperuricemia (AH), resulting from reduced uric acid excretion in the urine^1^. Without intervention, this condition progresses to a chronic state, leading to the accumulation of monosodium urate crystals in various body tissues, notably in the joints and kidneys^2^.

The buildup of monosodium urate in tissues has been linked to detrimental metabolic alterations observed in arterial hypertension, cardiovascular disease, and kidney disease^3^. This phenomenon initiates targeted pro-oxidant and pro-inflammatory processes that activate the renin-angiotensin-aldosterone system (RAAS) while suppressing endothelial nitric oxide release, leading to vasoconstriction and elevated glomerular blood pressure. Experimental investigations indicate that these mechanisms play an active role in the development of kidney injury and the progression of CKD^4^.

Between 85–90% of individuals with hyperuricemia harbor asymptomatic deposits of monosodium urate crystals in their tissues^5^. AH is characterized by elevated serum uric acid levels, exceeding 7.0 mg/dL for men and 6 mg/dL for women, devoid of any manifestations of gout or kidney stones^6^.

AH is acknowledged as an independent risk factor for both the onset and advancement of CKD, presenting a potentially modifiable aspect, as evidenced by various studies^7,8^. While urate-lowering therapy (ULT) is not typically recommended for asymptomatic hyperuricemia^9^, it is often prescribed to lower urate levels in CKD patients, weighing the risks and benefits of treatment^10^. Xanthine oxidase inhibitors, primarily allopurinol, are the cornerstone of ULT, impeding uric acid formation and reducing serum monosodium urate levels, and are commonly utilized in Brazil for managing elevated uric acid levels^11^.

Allopurinol’s ability to lower uric acid levels has been recognized as a valuable renoprotective strategy, aiding in the restoration of endothelial function, prevention of metabolic acidosis, and slowing of CKD progression^12,13^. The potential clinical advantages of ULT in mitigating early kidney damage and delaying CKD advancement have garnered significant attention from clinicians and nephrologists, prompting a surge in research activity in this field over the past two decades^14^. The elucidation of the outcomes of ULT holds the potential to shift the treatment paradigm toward actively managing asymptomatic hyperuricemia for the benefit of people with kidney disease^15^. Thus, this study aimed to assess whether administering allopurinol to people with chronic kidney disease and asymptomatic hyperuricemia enhances kidney function and delays progression to kidney failure.

## Methods

This retrospective cohort study utilized data retrieved from medical records housed at a single specialized facility, namely the Chronic Kidney Outpatient Clinic within the Nephrology Service of *Hospital São Sebastião Mártir* in Venâncio Aires, located in the *Vale do Rio Pardo* Health Region, Rio Grande do Sul, Brazil. The study spanned from 2006 to 2020. A total of eighty patients with a diagnosis of CKD and asymptomatic hyperuricemia were located (identified) in the medical records and divided into two groups: the case group, composed of 40 patients who received allopurinol at doses ranging from 100 to 300 mg/day, and the control group, consisting of 40 patients who did not receive allopurinol or any other medication for the treatment of hyperuricemia. The patients were followed by six different nephrologists, all of whom were aware of the controversies surrounding uric acid–lowering therapy. The specific criterion used to separate the groups was to respect each physician’s clinical decision to prescribe or not prescribe allopurinol and then to follow renal outcomes in these patients, regardless of the individual motivations for that decision.

The inclusion criteria were: 1) patients of both sexes aged 18 years or older, with an estimated glomerular filtration rate (eGFRcr) ranging from less than 60 mL/min/1.73 m^2^ to more than 15 mL/min/1.73 m^2^, and serum uric acid levels above 7.0 mg/dL for men and 6.0 mg/dL for women; 2) outpatient follow-up for a period of 24 months with four scheduled visits (T0, T1, T2, and T3), where T0 represented the initial visit, performed before any treatment for hyperuricemia was initiated; 3) adequately documented dosage and duration of treatment in both the intervention and control groups; 4) use of angiotensin-converting enzyme inhibitors (ACEIs) or angiotensin II receptor blockers (ARBs) for the treatment of hypertension, cardiovascular disease, or proteinuria; and 5) use of antidiabetic agents, lipid-lowering drugs, diuretics, and other medications necessary for optimal management of underlying conditions.

Exclusion criteria were: 1) individuals with gout, kidney stones, or liver disease; 2) those with incomplete baseline data, particularly missing serum uric acid levels; 3) patients with acute kidney injury or those requiring dialysis; and 4) patients receiving sodium-glucose cotransporter-2 (SGLT2) inhibitors or non-steroidal mineralocorticoid receptor antagonists (nsMRA), both considered renoprotective medications, which were not universally available in the public health system during the study period.

At the outset of the follow-up period and during subsequent study consultations, patients were categorized into stages of CKD based on their eGFRcr levels: stage 3a (59–45 mL/min/1.73 m^2^), stage 3b (44–30 mL/min/1.73 m^2^), and stage 4 (29–15 mL/min/1.73 m^2^), which corresponded to mild, moderate, and severe stages of kidney damage, respectively.

Demographic variables such as age, sex, ethnicity, and weight, along with clinical factors including the primary cause of kidney disease (such as hypertension, diabetes, or other causes), adherence to a purine-restricted diet, use of medications inhibiting urinary uric acid excretion, medications enhancing urinary uric acid excretion, serum uric acid levels, serum creatinine levels, hemoglobin levels, pyruvic transaminase levels, and 24-hour proteinuria, were evaluated. It was clearly observed in both groups that the strategies available during the study period for controlling comorbidities were equally optimized by the nephrologists. The eGFRcr was calculated using the 4-variable CKD-EPI equation incorporating age, sex, ethnicity, and serum creatinine levels during each consultation. This study received approval from the Institutional Review Board of the University of Santa Cruz do Sul (UNISC) (CAAE: 24394019.0.0000.5343; Opinion: 3.713.843). Declarations regarding Human Ethics and Consent to Participate were deemed not applicable due to the retrospective nature of the study.

Data were analyzed to examine factors associated with the progression of CKD in patients treated with allopurinol compared to those not receiving the drug. Sampling data underwent statistical analysis using IBM SPSS version 20.0 (IBM Corp., Armonk, New York, USA) for Windows 7. Values were presented as means and standard deviations. ANOVA for repeated measures with two factors, within-subjects (consultations) and between-subjects (control group vs. allopurinol group), was performed with Bonferroni adjustments. Categorical data were compared using the chi-square test, while quantitative variables were assessed using Student’s t-test. Statistical significance was defined as p < 0.05.

## Results

During the study period, 257 patients presenting with asymptomatic hyperuricemia were evaluated at the chronic kidney clinic. Among them, 177 patients who did not meet all inclusion criteria were excluded from the study. Consequently, our analysis focused on 80 patients (41 men and 39 women), with a mean age of 64.36 ± 13.6 years. Initial consultation (T0) data revealed several significant findings: the control group exhibited a lower mean body weight and standard deviation (SD) compared to the allopurinol group (76.4 ± 14.6 kg vs. 88.4 ± 19.1 kg; p = 0.003). Furthermore, the control group demonstrated significantly lower mean serum uric acid levels compared to the allopurinol group (7.40 ± 0.94 mg/dL vs. 8.52 ± 1.45 mg/dL; p < 0.001), as well as lower mean serum creatinine levels (p = 0.013) and higher mean eGFRcr values (p = 0.028). While the difference was not statistically significant, the control group exhibited a trend towards lower 24-hour proteinuria levels (p = 0.079) and higher mean hemoglobin levels (p = 0.015). There were no notable differences in mean pyruvic transaminase levels between the groups. In terms of primary etiology, 42 patients (52.5%) had hypertension, while 22 patients (27.5%) had diabetes (Table 1).

**Table 1:** Clinical and epidemiological characterization of the study population by group

Characteristics	Control group	Allopurinol group	Total	*p*
	N = 40	N = 40	N = 80	
Sex, n (%)				
Male	18 (45)	23 (57.5)	41 (51.25)	0.263^****^
Female	22 (55)	17 (42.5)	39 (48.75)	
Age^*^	63.82 ± 14.98	64.90 ± 12.37	64.36 ± 13.66	0.727^***^
Weight (Kg)^*^	76.46 ± 14.65	88.42 ± 19.12	82.52 ± 17.98	0.003^***^
Low-purine diet, n (%)				
Yes	28 (70)	21 (52.5)	49 (61.3)	0.108^****^
No	12 (30)	19 (47.5)	31 (38.7)	
Serum uric acid (mg/dL)^*^	7.40 ± 0.94	8.52 ± 1.45	7.99 ± 1.33	< 0.001^***^
Serum creatinine (mg/dL)^*^	1.64 ± 0.39	1.92 ± 0.48	1.80 ± 0.45	0.013^***^
eGFRcr (mL/min/1.73 m^2^)^*^	52.53 ± 18.29	40.78 ± 15.65	48.09 ± 18.06	0.028^***^
24-hour proteinuria (mg/24h)^*^	287.31 ± 436.64	762.53 ± 910.76	549.5 ± 764.15	0.079^***^
Serum hemoglobin (g/dL)^*^	13.24 ± 1.86	12.02 ± 1.49	12.58 ± 1.76	0.015^***^
Pyruvic transaminase (U/L)^*^	20.20 ± 10.07	17.67 ± 4.71	19.00 ± 7.89	0.488^***^
Primary cause of the disease, n (%)				
Hypertension	21 (52.5)	21 (52.5)	42 (52.5)	0.806^****^
Diabetes	10 (25.0)	12 (30.0)	22 (27.5)	
Others^**^	9 (22.5)	7 (17.5)	16 (20.0)	

Notes – *Mean ± standard deviation of the data collected at the initial consultation (T0).

**Heart failure, hyperlipidemia, nephritis, etc.

***p-value calculated using Student’s t-test.

****p-value calculated using the chi-square test.

**Figure 1:**
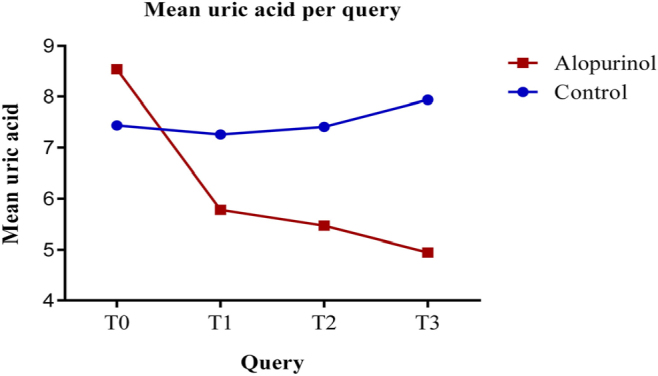
Serum uric acid (mg/dL): mean per group.

The mean ± SD of uric acid levels increased in the control group during the follow-up period. However, there were no statistically significant differences between the consultations. At T0, the mean uric acid level was 7.40 ± 0.943 mg/dL; at T1, it was 7.23 ± 1.016 mg/dL; at T2, it was 7.44 ± 1.177 mg/dL; and at T3, it was 7.87 ± 1.456 mg/dL (p = 0.081) (Figure 1A). In the allopurinol group, the serum uric acid levels ± SD were consistently lower at each review consultation during the follow-up period. Specifically, at T0, the mean uric acid level was 8.52 ± 1.455 mg/dL; at T1, it decreased to 5.78 ± 1.422 mg/dL; at T2, it further decreased to 5.52 ± 1.112 mg/dL; and at T3, it reached the lowest value of 4.97 ± 1.102 mg/dL. In this group, there were statistically significant differences in mean uric acid levels between the following consultations: T0–T1, T0–T2, and T0–T3 (p < 0.001), as well as between T1–T3 (p = 0.005). However, there were no significant differences between T1–T2 (p = 1.000) and T2–T3 (p = 0.081) (Figure 1A).

In the control group, there was a notable decrease in the mean estimated glomerular filtration rate (eGFRcr) ± SD over the 24-month follow-up period. Specifically, at T0, the mean eGFRcr was 40.41 ± 11.91 mL/min/1.73 m^2^, decreasing to 37.64 ± 10.604 mL/min/1.73 m^2^ at T1, to 33.38 ± 10.019 mL/min/1.73 m^2^ at T2, and eventually to 29.80 ± 9.762 mL/min/1.73 m^2^ at T3. For the control group, there was a statistically significant difference between the mean eGFR values throughout all consultations (p < 0.001) (Figure 1).

**Figure 2:**
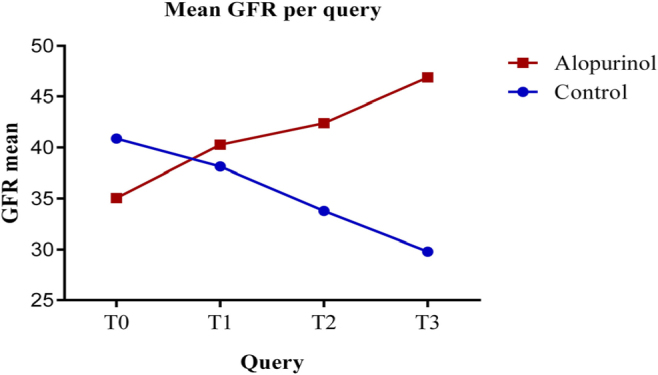
Estimated glomerular filtration rate (mL/min/1.73 m^2^): mean per group.

In the allopurinol group, the mean ± SD eGFRcr showed a consistent increase across all review consultations. Specifically, at T0, the mean eGFR was 35.06 ± 11.067 mL/min/1.73 m^2^, increasing to 40.28 ± 11.480 mL/min/1.73 m^2^ at T1, to 42.41 ± 12.098 mL/min/1.73 m^2^ at T2, and reaching 46.89 ± 14.568 mL/min/1.73 m^2^ at T3. In this group, there was a notable difference in statistical significance between consultations. Although no statistically significant change was observed between T1 and T2 (p = 0.215), all other consultations reached significant differences, with p < 0.001 (Figure 2).

In the control group, the classification of patients based on CKD stage across successive consultations revealed a significant increase in the number of patients in stages 3b and 4 by the end of the follow-up period. Notably, four patients, all female, progressed to stage 5 CKD at the conclusion of the 24-month period. Conversely, in the allopurinol group, a contrasting trend was observed, with a higher proportion of patients remaining in stages 2, 3a, and 3b. None of the patients in this group progressed to stage 5 CKD by the end of the 24-month treatment period (p < 0.001). The mean dosage of allopurinol ± SD at consultation T1 was 207.5 ± 99.71 mg; at T2, 212.5 ± 96.57 mg; and at T3, it was 205.0 ± 98.58 mg. No adverse events associated with the use of allopurinol were reported during the analysis (Table 2).

**Table 2:** Staging of the study population throughout follow-up per group

Characteristics	Control group	Allopurinol group	Total	p^**^
	N = 40	N = 40	N = 80 (100%)	
CKD stage at consultation T0, n (%)^*^				
3a	16 (40.0)	8 (20.0)	24 (30.0)	0.062
3b	17 (42.5)	17 (42.5)	34 (42.5)	
4	7 (17.5)	15 (37.5)	22 (27.5)	
CKD stage at consultation T1, n (%)^*^				
2	1 (2.5)	1 (2.5)	2 (2.5)	0.494
3a	9 (22.5)	15 (37.5)	24 (30.0)	
3b	23 (57.5)	17 (42.5)	40 (50.5)	
4	7 (17.5)	7 (17.5)	14 (17.5)	
CKD stage at consultation T2, n (%)^*^				
2	0 (0.0)	3 (7.5)	3 (3.8)	0.015
3a	5 (12.5)	15 (37.5)	20 (25.0)	
3b	21 (52.5)	15 (37.5)	36 (45.0)	
4	12 (17.5)	7 (17.5)	19 (23.8)	
5	2 (5.0)	0 (0.0)	2 (2.5)	
CKD stage at consultation T3, n (%)^*^				
2	0 (0.0)	8 (20.0)	8 (10.1)	<0.001
3a	3 (7.7)	13 (37.5)	16 (20.3)	
3b	15 (38.5)	13 (42.5)	28 (35.4)	
4	17 (43.6)	6 (17.5)	23 (29.1)	
5	4 (10.3)	0 (0.0)	4 (5.1)	

Notes – *CKD stages according to eGFRcr (mL/min/1.73 m^2^): 2 (60–89); 3a (45–59); 3b (30–44); 4 (15–29); 5 (< 15).

**p-value calculated using the chi-square test.

## Discussion

In this study, asymptomatic hyperuricemia was found to be correlated with a decline in kidney function, indicating that elevated uric acid levels may contribute to kidney damage. This association was initially observed in the control group, where there was an unfavorable progression of CKD. Subsequently, the clinical benefit of ULT was demonstrated in the allopurinol group. By reducing uric acid levels, allopurinol treatment led to an improvement in kidney function. This suggests that lowering uric acid levels may play a role in preserving or enhancing kidney function.

In the control group, it was noted that the standard treatment protocol for asymptomatic hyperuricemia fell short in managing uric acid levels, with serum uric acid increasing steadily during follow-up consultations. Concurrently, the mean eGFRcr declined progressively, leading to the reclassification of patients into worsening stages of kidney disease. Remarkably, within 24 months, four female subjects from the control group experienced a decline in kidney function to the point of requiring dialysis-based renal replacement therapy (RRT). The persistence of elevated uric acid levels has been linked to a heightened risk of reaching end-stage kidney disease^16^. Furthermore, research has highlighted a correlation between hyperuricemia and sex, indicating a greater likelihood of women experiencing diminished eGFRcr due to urate deposition. Hyperuricemia emerges as a potential independent predictor of end-stage kidney disease in females^17^.

Among the 40 patients who underwent ULT with allopurinol, a notable reduction in mean serum uric acid levels was observed over the course of 24 months, affirming the efficacy of allopurinol in decreasing serum uric acid among individuals with CKD and eGFRcr levels ranging from 60 to 15 mL/min/1.73 m^2^. Furthermore, several studies have consistently demonstrated the sustained and long-term benefits of allopurinol in lowering uric acid levels and conferring advantages to CKD patients^1,18^.

In patients who received continuous allopurinol therapy, there was a consistent increase in mean eGFR at each follow-up visit, leading to the reclassification of patients into milder stages of kidney disease. Notably, none of the individuals in this group progressed to the stage requiring dialysis within the 24-month period, highlighting the sustained clinical advantages of ULT with allopurinol for kidney outcomes in this specific population.

Numerous studies have consistently demonstrated improvements in kidney function in patients undergoing ULT with allopurinol, both within treatment groups and in comparison with control groups^1,16,19^. Golmohammadi et al.^1^, for instance, reported compelling findings demonstrating a substantial reduction in serum uric acid levels, decreased serum creatinine levels, and improved eGFR among individuals with mild CKD after 12 months of allopurinol use. Similarly, Goicoechea et al.^16^ reported an improvement in estimated glomerular filtration rate following 24 months of allopurinol therapy. In a subsequent study in 2015, the same authors demonstrated consistent outcomes with allopurinol treatment for up to 84 months, concluding that prolonged therapy may mitigate the rate of progression^20^. Moreover, a systematic review of randomized controlled trials conducted in 2017 suggested that xanthine oxidase inhibitors may offer direct kidney benefits beyond their hypouricemic effects. Within this review, trials utilizing allopurinol exhibited a deceleration in eGFR decline over time and a reduction in the risk of reaching end-stage kidney disease^19^.

Elevated serum urate and urinary uric acid levels have been implicated in adverse kidney events and may contribute to the progression of CKD, although the precise mechanism underlying kidney injury remains elusive. Pathophysiological investigations in animal models have revealed that hyperuricemia triggers inflammatory processes that promote vasoactive responses, sodium retention, vascular constriction, and elevated blood pressure^21^.

Experimental evidence suggests that uric acid serves as a potent activator of the RAAS in humans^22^, in addition to activating other crucial vasoconstrictors such as endothelin and thromboxane, while concurrently inhibiting vasodilator pathways, including nitric oxide^23^. Moreover, elevated uric acid levels promote intracellular oxidative stress, leading to inflammation, cellular proliferation, and renal fibrosis^4^. Furthermore, uricosuria and the presence of urate crystals can induce tubular damage through direct mechanisms or by triggering tubulointerstitial inflammation^24^.

The impact of ULT with allopurinol on the progression of CKD remains an area of ongoing research. Experimental studies conducted in rats have demonstrated that allopurinol effectively prevents the onset of hypertension and modulates renin and nitric oxide levels, thereby mitigating the hypertensive effects associated with hyperuricemia^21^. In a study by Yelken et al.^25^, it was shown that allopurinol treatment for hyperuricemia reduced oxidative stress, ameliorated endothelial dysfunction, and improved kidney function in CKD patients. Another study conducted by Bayram et al.^12^ explored metabolic acidosis as a risk factor for CKD progression and found that allopurinol administration led to a decrease in uric acid levels and an increase in serum bicarbonate, which could potentially aid in preventing acidemia and slowing the progression of kidney disease.

By inhibiting xanthine oxidase, an enzyme responsible for producing superoxide, allopurinol exerts a crucial antioxidant effect, curbing the generation of oxygen-free radicals and deactivating pro-inflammatory pathways implicated in CKD progression^26^. These findings collectively suggest that allopurinol may hold promise as a therapeutic intervention to mitigate CKD progression, although further research is warranted to elucidate its precise mechanisms and therapeutic efficacy.

All 80 patients in our study received standard care for CKD and asymptomatic hyperuricemia. Consequently, we inferred that the improvement in kidney function observed in the allopurinol group stemmed from the reduction in uric acid levels through ULT. Throughout our research, the administration of allopurinol, averaging close to 200 mg per day, effectively lowered uric acid levels to below 5 mg/dL by the end of the follow-up period. This mean uric acid result of approximately 5 mg/dL may indicate a potentially beneficial target level for preserving kidney function^27^ or may suggest lower cutoff values for defining asymptomatic hyperuricemia than those traditionally used^28^.

Assessing the impact of hyperuricemia in individuals with CKD poses a considerable challenge due to its complexity. The observed negative association between asymptomatic hyperuricemia and CKD may have been influenced by various other risk factors present in our population, including age, weight, diet, hypertension, diabetes, medications affecting uric acid excretion, and proteinuria. Notably, the mean age of the participants exceeded 60 years, with no discernible differences between the case and control groups. Considering that kidney function declines with age due to the progressive loss of nephrons, population aging emerges as a significant risk factor for CKD^28^. Nevertheless, research indicates that elevated serum uric acid levels in both elderly and middle-aged populations correlate negatively with eGFR^29^.

Patients in the allopurinol group exhibited significantly higher body weights compared to those in the control group, potentially aligning with findings from other studies suggesting a direct association between overweight and obesity and serum uric acid levels^30,31^. Among all patients in our study, 52.5% had hypertension and 22% had diabetes as the primary cause of CKD, both recognized as risk factors for progressive kidney damage and potentially confounding variables in our population. Nonetheless, multiple studies have established a direct link between elevated uric acid levels and an increased risk of hypertension and diabetes^4,21^.

Recently, two studies evaluated the efficacy of allopurinol in halting CKD progression, but neither study demonstrated significant clinical benefits for patients treated with the drug^32,33^. Importantly, these studies acknowledged that some participants did not initially present with hyperuricemia; thus, a portion of the subjects had normal serum uric acid levels. In contrast, our retrospective observational study exclusively enrolled patients with asymptomatic hyperuricemia, ensuring consistency throughout the 24-month follow-up period without any losses to follow-up.

Another study evaluated the biological mechanisms triggered by elevated soluble uric acid and crystalline uric acid separately, discussing the role of asymptomatic hyperuricemia in CKD, CVD, and sterile inflammation, pointing to possibilities for the use of uric acid-lowering therapy in patients with kidney disease, especially when uric acid crystals are identified in the urine^34^. Although many current guidelines, such as KDIGO 2024^35^, do not recommend routine treatment of asymptomatic hyperuricemia to mitigate CKD progression due to limited evidence, the debate has persisted for years, supported by conflicting experimental models and clinical data suggesting a pathogenic role of uric acid in the undesirable decline in renal function. Recent studies have questioned the potential benefit of treating asymptomatic hyperuricemia in slowing CKD progression. The FIX-CKD randomized clinical trial, one of the most robust trials conducted to date, showed no effect of allopurinol on attenuating the decline in kidney function^33^. In addition, meta-analyses published in recent years have reported inconsistent results and substantial heterogeneity across studies related to differences in inclusion criteria, population characteristics, comorbidity control, and follow-up duration. This methodological variability hampers the ability to draw definitive conclusions and reinforces the need for well-designed randomized controlled trials with standardized clinical variables and long-term follow-up^36,37^.

A limitation in our study was the inclusion of medications that modify uric acid excretion, in particular diuretics, low-dose acetylsalicylic acid, certain antihypertensives, and lipid-lowering drugs^38,39^. Although not statistically significant, the utilization of these medications may have influenced serum uric acid levels, and this aspect was not examined in isolation. Additionally, it is important to emphasize that we were unable to obtain the body mass index (BMI) of the patients due to the absence of appropriate height-measuring instruments in the outpatient clinic. It is noteworthy that the patients received care through the public healthcare system, which is the prevailing system in the country. The significance of proteinuria as a risk factor for CKD progression is widely acknowledged^29^. However, in our study, the patients evaluated did not use antiproteinuric medications from the SGLT2 inhibitor and nsMRA classes. We did not analyze the influence of proteinuria on CKD, which could potentially introduce bias. It is worth noting that although proteinuria was not statistically significant when comparing the two groups, its omission from our analysis should be considered. Additionally, the length of follow-up and the sample size may have also limited the results presented. Another important limitation of this study concerns the potential influence of comorbidities such as hypertension, diabetes, and excess body weight, which are highly prevalent among individuals with CKD and may act as confounding factors in the relationship between hyperuricemia and kidney function. These conditions have pathophysiological mechanisms that affect both serum uric acid levels and glomerular filtration rate, making it difficult to determine a direct causal effect. Although we described these variables and compared their distribution between groups, the absence of systematic control or statistical adjustment for these factors prevents definitive conclusions regarding the independence of the observed association. Therefore, our findings should be interpreted with caution, underscoring the need for prospective studies with standardized collection of comorbidities and potential confounders to more robustly elucidate the role of asymptomatic hyperuricemia in CKD progression^27,40^.

We concur that additional randomized, placebo-controlled clinical trials are imperative to draw definitive conclusions regarding the heightened risk of CKD progression associated with asymptomatic hyperuricemia, as well as the efficacy and safety of utilizing allopurinol to lower serum uric acid levels and mitigate the decline in GFR across various stages of CKD. Finally, it is worth highlighting that the study population, based in Brazil, consists of patients treated within the Unified Health System (*Sistema Único de Saúde* – SUS), a publicly funded healthcare system accessible to low-income populations. It is important to emphasize that allopurinol treatment is a significant tool provided free of charge to patients with CKD and hyperuricemia, underscoring the importance of this pharmacological strategy.

## Conclusion

In this study, we determined that allopurinol-based urate-lowering therapy constitutes a valuable approach for reducing serum uric acid levels and enhancing kidney function among patients with chronic kidney disease and asymptomatic hyperuricemia during a 24-month outpatient follow-up period. The administered doses of allopurinol were both safe and effective, consistently resulting in increased eGFRcr at each review visit and aiding in delaying progression to kidney failure. Our findings reinforce the importance of considering ULT with allopurinol for outpatients with chronic kidney disease (CKD) and asymptomatic hyperuricemia, following appropriate guidance and follow-up through regular consultations throughout the entire period.

## Data Availability

The datasets generated and/or analyzed during the current study are available from the corresponding author on reasonable request.
