# Supplementary material for: Assessment of allopurinol use in patients with chronic kidney disease and asymptomatic hyperuricemia: a retrospective cohort study
Source: J Bras Nefrol. 2026 Apr 20;48(3):e20250243. doi: 10.1590/2175-8239-JBN-2025-0243en (PMC13101826; doi:10.1590/2175-8239-JBN-2025-0243en)
Supplement: Translation

# Translation: Avaliação do uso de alopurinol em pacientes com doença renal crônica e hiperuricemia assintomática: um estudo de coorte retrospectivo

**DOI:** 10.1590/2175-8239-JBN-2025-0243pt

**Authors:** Maria Elaine Latosinski Santos de Souza, Tiago Antônio Heringer, Ana Paula Helfer Schneider, Lia Gonçalves Possuelo, Andreia Rosane de Moura Valim

**Affiliations:** 1 Universidade de Santa Cruz do Sul, Santa Cruz do Sul, RS, Brasil.

**Keywords:** Ácido Úrico, Alopurinol, Hiperuricemia, Insuficiência Renal Crônica

## Abstract

**Introdução:**

A hiperuricemia assintomática (HA) é comum em pacientes com doença renal crônica (DRC) e tem sido apontada como uma condição modificável, cuja abordagem poderia ampliar as possibilidades de prevenção e tratamento da lesão renal.

**Objetivo:**

Avaliar se a administração de alopurinol em pessoas com doença renal crônica e hiperuricemia assintomática melhora a função renal e retarda a progressão para insuficiência renal.

**Métodos:**

Este é um estudo de coorte retrospectivo, com coleta de dados de prontuários médicos em um centro especializado, entre 2006 e 2020. Oitenta indivíduos nos estágios 3 e 4 da DRC, com HA, foram divididos em dois grupos: 40 pacientes receberam alopurinol e 40 não receberam medicação. Os pacientes foram acompanhados por 24 meses, com quatro consultas. Os níveis séricos de ácido úrico e a taxa de filtração glomerular estimada com base na creatinina (eTFGcr), calculada pela fórmula CKD-EPI, foram comparados intra e intergrupos utilizando média, desvio padrão e análise de variância (ANOVA).

**Resultados:**

Oitenta pacientes foram incluídos no estudo. Ao comparar 40 pacientes sem tratamento para HA com 40 pacientes em terapia hipouricemiante (THU) com alopurinol, observouse que a média do ácido úrico sérico diminuiu em todas as consultas de acompanhamento no grupo alopurinol, além de um aumento significativo na taxa de filtração glomerular estimada (eTFGcr) média após o início do tratamento (p < 0,001). Nenhum paciente do grupo alopurinol desenvolveu doença renal terminal (DRT) em 24 meses (p < 0,001). Resultados semelhantes não foram observados no grupo controle.

**Conclusão:**

O alopurinol mostrou-se útil na redução dos níveis de ácido úrico sérico, na melhora da função renal e no retardo da progressão para insuficiência renal em pacientes com HA.

## Introdução

Pacientes diagnosticados com doença renal crônica (DRC) e manejados em regime ambulatorial frequentemente apresentam hiperuricemia assintomática (HA), resultante da redução da excreção de ácido úrico pela urina^1^. Sem intervenção, essa condição pode progredir para um estado crônico, levando ao acúmulo de cristais de urato monossódio em vários tecidos do corpo, notadamente nas articulações e nos rins^2^.

O acúmulo de urato monossódio nos tecidos tem sido associado a alterações metabólicas prejudiciais observadas na hipertensão arterial, nas doenças cardiovasculares e na doença renal^3^. Esse fenômeno desencadeia processos pró-oxidantes e pró-inflamatórios direcionados, que ativam o sistema renina-angiotensina-aldosterona (SRAA), ao mesmo tempo em que suprimem a liberação de óxido nítrico endotelial, levando à vasoconstrição e ao aumento da pressão arterial glomerular. Investigações experimentais indicam que esses mecanismos desempenham um papel ativo no desenvolvimento de lesões renais e na progressão da DRC^4^.

Entre 85% e 90% dos indivíduos com hiperuricemia possuem depósitos assintomáticos de cristais de urato monossódio em seus tecidos^5^. A HA é caracterizada por níveis elevados de ácido úrico sérico, acima de 7,0 mg/dL para homens e de 6 mg/dL para mulheres, na ausência de quaisquer manifestações de gota ou cálculo renal^6^.

A HA é reconhecida como um fator de risco independente tanto para o início quanto para a progressão da DRC, apresentando um aspecto potencialmente modificável, como evidenciado por diversos estudos^7,8^. Embora a terapia redutora de urato (TRU) não seja tipicamente recomendada para a hiperuricemia assintomática^9^, ela é frequentemente prescrita para reduzir os níveis de urato em pacientes com DRC, ponderando-se os riscos e benefícios do tratamento^10^. Inibidores da xantina oxidase, principalmente o alopurinol, são a base da TRU, impedindo a formação de ácido úrico e diminuindo os níveis séricos de urato monossódio, sendo comumente utilizados no Brasil para o manejo de níveis elevados de ácido úrico^11^.

A capacidade do alopurinol de reduzir os níveis de ácido úrico tem sido reconhecida como uma valiosa estratégia renoprotetora, auxiliando na restauração da função endotelial, na prevenção da acidose metabólica e na desaceleração da progressão da DRC^12,13^. As potenciais vantagens clínicas da TRU em mitigar danos renais precoces e retardar a progressão da DRC têm atraído atenção significativa de clínicos e nefrologistas, provocando um aumento na atividade de pesquisa neste campo ao longo das últimas duas décadas^14^. A elucidação dos resultados da TRU possui o potencial de mudar o paradigma de tratamento em direção ao manejo ativo da hiperuricemia assintomática em benefício das pessoas com doença renal^15^. Assim, este estudo teve como objetivo avaliar se a administração de alopurinol em pessoas com doença renal crônica e hiperuricemia assintomática melhora a função renal e retarda a progressão para a insuficiência renal.

## Métodos

Este estudo de coorte retrospectivo utilizou dados extraídos de prontuários médicos armazenados em uma única instituição especializada, nomeadamente a Clínica Ambulatorial de Doença Renal Crônica do Serviço de Nefrologia do Hospital São Sebastião Mártir, em Venâncio Aires, localizado na Região de Saúde do Vale do Rio Pardo, Rio Grande do Sul, Brasil. O estudo abrangeu o período de 2006 a 2020. Um total de oitenta pacientes com diagnóstico de DRC e hiperuricemia assintomática foi localizado (identificado) nos prontuários médicos e dividido em dois grupos: o grupo caso, composto por 40 pacientes que receberam alopurinol em doses variando de 100 a 300 mg/dia, e o grupo controle, composto por 40 pacientes que não receberam alopurinol ou qualquer outro medicamento para o tratamento da hiperuricemia. Os pacientes foram acompanhados por seis nefrologistas diferentes, todos cientes das controvérsias em torno da TRU. O critério específico utilizado para separar os grupos foi o respeito à decisão clínica de cada médico em prescrever ou não prescrever alopurinol, e então acompanhar os desfechos renais nesses pacientes, independentemente das motivações individuais para essa decisão.

Os critérios de inclusão foram: 1) pacientes de ambos os sexos, com 18 anos ou mais, com taxa de filtração glomerular estimada (TFGe) variando de menos de 60 mL/min/1,73 m^2^ a mais de 15 mL/min/1,73 m^2^, e níveis séricos de ácido úrico acima de 7,0 mg/dL para homens e de 6,0 mg/dL para mulheres; 2) acompanhamento ambulatorial por um período de 24 meses, com quatro visitas agendadas (T0, T1, T2 e T3), em que T0 representou a visita inicial, realizada antes do início de qualquer tratamento para hiperuricemia; 3) dosagem e duração do tratamento adequadamente documentadas em ambos os grupos, intervenção e controle; 4) uso de inibidores da enzima conversora de angiotensina (IECA) ou bloqueadores do receptor da angiotensina II (BRA) para tratamento de hipertensão, doença cardiovascular ou proteinúria; e 5) uso de agentes antidiabéticos, medicamentos redutores de lipídios, diuréticos e outros fármacos necessários para o manejo ideal das condições subjacentes.

Os critérios de exclusão foram: 1) indivíduos com gota, cálculo renal ou doença hepática; 2) aqueles com dados basais incompletos, particularmente na ausência de níveis séricos de ácido úrico; 3) pacientes com lesão renal aguda ou que necessitavam de diálise; e 4) pacientes recebendo inibidores do cotransportador de sódio-glicose-2 (SGLT2) ou antagonistas do receptor mineralocorticoide não esteroides (nsMRA), ambos considerados medicamentos renoprotetores, que não estavam universalmente disponíveis no sistema público de saúde durante o período do estudo.

No início do período de acompanhamento e durante as consultas subsequentes do estudo, os pacientes foram categorizados em estágios de DRC com base em seus níveis de TFGe: estágio 3a (59–45 mL/min/1,73 m^2^), estágio 3b (44–30 mL/min/1,73 m^2^) e estágio 4 (29–15 mL/min/1,73 m^2^), correspondendo a estágios leve, moderado e grave de dano renal, respectivamente.

Variáveis demográficas, como idade, sexo, etnia e peso, bem como fatores clínicos, incluindo a causa primária da doença renal (como hipertensão, diabetes ou outras causas), adesão a uma dieta restrita em purinas, uso de medicamentos que inibem a excreção urinária de ácido úrico, medicamentos que aumentam a excreção urinária de ácido úrico, níveis séricos de ácido úrico, níveis séricos de creatinina, níveis de hemoglobina, níveis de transaminases pirúvicas e proteinúria de 24 horas, foram avaliados. Observouse em ambos os grupos estudados que as estratégias para controlar comorbidades, disponíveis durante o período do estudo, foram igualmente otimizadas pelos nefrologistas. A eTFGcr foi calculada usando a equação CKD-EPI de quatro variáveis, incorporando idade, sexo, etnia e níveis séricos de creatinina durante cada consulta. Este estudo recebeu aprovação do Comitê de Ética Institucional da Universidade de Santa Cruz do Sul (UNISC) (CAAE: 24394019.0.0000.5343; Parecer: 3.713.843). As declarações sobre Ética Humana e Consentimento para Participar foram consideradas não aplicáveis, devido à natureza retrospectiva do estudo.

Os dados foram analisados para examinar os fatores associados à progressão da DRC em pacientes tratados com alopurinol, em comparação com aqueles que não receberam o medicamento. Os dados de amostragem foram submetidos à análise estatística utilizando o IBM SPSS versão 20.0 (IBM Corp., Armonk, Nova York, EUA) para Windows 7. Os valores foram apresentados como média e desvio padrão. A ANOVA para medidas repetidas com dois fatores, intra-sujeitos (consultas) e entre sujeitos (grupo controle vs. grupo alopurinol), foi realizada com ajustes de Bonferroni. Dados categóricos foram comparados usando o teste qui-quadrado, enquanto variáveis quantitativas foram avaliadas usando o teste t de Student. A significância estatística foi definida como p < 0,05.

## Resultados

Durante o período do estudo, 257 pacientes com hiperuricemia assintomática foram avaliados na clínica de doença renal crônica. Dentre eles, 177 pacientes que não preencheram todos os critérios de inclusão foram excluídos do estudo. Consequentemente, nossa análise focou em 80 pacientes (41 homens e 39 mulheres), com idade média de 64,36 ± 13,6 anos. Os dados da consulta inicial (T0) revelaram várias descobertas significativas: o grupo controle apresentou peso corporal médio e desvio padrão (DP) mais baixos em comparação ao grupo que recebeu alopurinol (76,4 ± 14,6 kg vs. 88,4 ± 19,1 kg; p = 0,003). Além disso, o grupo controle demonstrou níveis médios de ácido úrico sérico significativamente mais baixos em comparação ao grupo alopurinol (7,40 ± 0,94 mg/dL vs. 8,52 ± 1,45 mg/dL; p < 0,001), bem como níveis médios de creatinina sérica mais baixos (p = 0,013) e valores médios de eTFGcr mais altos (p = 0,028). Embora a diferença não tenha sido estatisticamente significativa, o grupo controle apresentou tendência a níveis mais baixos de proteinúria de 24 horas (p = 0,079) e níveis médios de hemoglobina mais altos (p = 0,015). Não houve diferenças notáveis nos níveis médios de transaminases pirúvicas entre os grupos. Em termos de etiologia primária, 42 pacientes (52,5%) apresentavam hipertensão, enquanto 22 (27,5%) tinham diabetes (Tabela 1).

**Tabela 1:** Caracterização clínica e epidemiológica da população do estudo por grupo

Características	Grupo controle	Grupo alopurinol	Total	*p*
	N = 40	N = 40	N = 80
Sexo, n (%)	18 (45)	23 (57,5)	41 (51,25)	0,263^****^
Masculino
Feminino	22 (55)	17 (42,5)	39 (48,75)	
Idade^*^	63,82 ± 14,98	64,90 ± 12,37	64,36 ± 13,66	0,727^***^
Peso (kg)^*^	76,46 ± 14,65	88,42 ± 19,12	82,52 ± 17,98	0,003^***^
Dieta com baixo teor de purina, n (%)
Sim	28 (70)	21 (52,5)	49 (61,3)	0,108^****^
Não	12 (30)	19 (47,5)	31 (38,7)	
Ácido úrico sérico (mg/dL)^*^	7,40 ± 0,94	8,52 ± 1,45	7,99 ± 1,33	<0,001^***^
Creatinina sérica (mg/dL)^*^	1,64 ± 0,39	1,92 ± 0,48	1,80 ± 0,45	0,013^***^
eTFGcr (mL/min/1,73 m^2^)^*^	52,53 ± 18,29	40,78 ± 15,65	48,09 ± 18,06	0,028
Proteinúria em 24 horas (mg/24h)^*^	287,31 ± 436,64	762,53 ± 910,76	549,5 ± 764,15	0,079^***^
Hemoglobina sérica (g/dL)^*^	13,24 ± 1,86	12,02 ± 1,49	12,58 ± 1,76	0,015
Transaminase pirúvica (U/L)^*^	20,20 ± 10,07	17,67 ± 4,71	19,00 ± 7,89	0,488
Causa primária da doença, n (%) Hipertensão	21 (52,5)	21 (52,5)	42 (52,5)	0,806^****^
Diabetes	10 (25,0)	12 (30,0)	22 (27,5)	
Outros^**^	9 (22,5)	7 (17,5)	16 (20,0)	

Notas – *Média ± desvio padrão dos dados coletados na consulta inicial (T0).

**Insuficiência cardíaca, hiperlipidemia, nefrite, etc.

***Valor p calculado utilizando o teste t de Student.

****Valor p calculado utilizando o teste qui-quadrado.

A média ± DP do ácido úrico aumentou no grupo controle durante o período de acompanhamento. No entanto, não houve diferenças estatisticamente significativas entre as consultas. Em T0, o nível médio de ácido úrico foi de 7,40 ± 0,943 mg/dL; em T1, foi de 7,23 ± 1,016 mg/dL; em T2, foi de 7,44 ± 1,177 mg/dL; e em T3, foi de 7,87 ± 1,456 mg/dL (p = 0,081) (Figura 1A). No grupo alopurinol, os níveis séricos de ácido úrico ± DP foram consistentemente menores em cada consulta de revisão durante o período de acompanhamento. Especificamente, em T0, o nível médio de ácido úrico foi de 8,52 ± 1,455 mg/dL; em T1, diminuiu para 5,78 ± 1,422 mg/dL; em T2, diminuiu ainda mais para 5,52 ± 1,112 mg/dL; e em T3, atingiu o valor mais baixo, de 4,97 ± 1,102 mg/dL. Neste grupo, houve diferenças estatisticamente significativas nos níveis médios de ácido úrico entre as seguintes consultas: T0-T1, T0-T2 e T0-T3 (p < 0,001), bem como entre T1-T3 (p = 0,005). No entanto, não houve diferenças significativas entre T1-T2 (p = 1,000) e T2-T3 (p = 0,081).

**Figura 1:** Ácido úrico sérico (mg/dL): média por grupo.

No grupo controle, observou-se uma redução significativa na taxa de filtração glomerular estimada (eTFGcr) média ± DP ao longo de 24 meses. Especificamente, em T0, a eTFGcr média foi de 40,41 ± 11,91 mL/min/1,73 m^2^, diminuindo para 37,64 ± 10,604 mL/min/1,73 m^2^ em T1, para 33,38 ± 10,019 mL/min/1,73 m^2^ em T2 e, finalmente, para 29,80 ± 9,762 mL/min/1,73 m^2^ em T3. Para o grupo controle, houve uma diferença estatisticamente significativa entre as médias da eTFGcr ao longo de todas as consultas, com p < 0,001 (Figura 1).

Já no grupo do alopurinol, a eTFGcr média ± DP apresentou um aumento consistente em todas as consultas de revisão. Especificamente, em T0, a eTFGcr média foi de 35,06 ± 11,067 mL/min/1,73 m^2^, aumentando para 40,28 ± 11,480 mL/min/1,73 m^2^ em T1, para 42,41 ± 12,098 mL/min/1,73 m^2^ em T2 e atingindo 46,89 ± 14,568 mL/min/1,73 m^2^ em T3. Neste grupo, houve uma diferença estatisticamente significativa entre as consultas de revisão. Além disso, embora nenhuma diferença significativa tenha sido observada entre as consultas T1 e T2 (p = 0,215), diferenças significativas foram encontradas entre as demais consultas, com p < 0,001 (Figura 2).

**Figura 2:** Taxa de filtração glomerular estimada (mL/min/1,73 m^2^):

No grupo controle, a classificação dos pacientes com base no estágio da DRC em consultas sucessivas revelou um aumento significativo no número de pacientes nos estágios 3b e 4 ao final do período de acompanhamento. Particularmente, quatro pacientes, todas do sexo feminino, progrediram para o estágio 5 da DRC ao término do período de 24 meses. Por outro lado, no grupo alopurinol, observou-se uma tendência oposta, com uma proporção maior de pacientes permanecendo nos estágios 2, 3a e 3b. É importante ressaltar que nenhum dos pacientes deste grupo progrediu para o estágio 5 da DRC ao final do período de tratamento de 24 meses (p < 0,001). A dose média de alopurinol ± DP na consulta T1 foi de 207,5 ± 99,71 mg; em T2, foi de 212,5 ± 96,57 mg; e em T3, foi de 205,0 ± 98,58 mg. Não foram relatados eventos adversos associados ao uso de alopurinol durante a análise (Tabela 2).

**Tabela 2:** Estadiamento da população do estudo ao longo do acompanhamento por grupo

Características	Grupo controle	Grupo alopurinol	Total	p^**^
	N = 40	N = 40	N = 80 (100%)	
Estágio da DRC na consulta T0, n (%)^*^			
3a	16 (40,0)	8 (20,0)	24 (30,0)	0,062
3b	17 (42,5)	17 (42,5)	34 (42,5)	
4	7 (17,5)	15 (37,5)	22 (27,5)	
Estágio da DRC na consulta T1, n (%)^*^			
2	1 (2,5)	1 (2,5)	2 (2,5)	0,494
3a	9 (22,5)	15 (37,5)	24 (30,0)	
3b	23 (57,5)	17 (42,5)	40 (50,5)	
4	7 (17,5)	7 (17,5)	14 (17,5)	
Estágio da DRC na consultaT2, n (%)^*^			
2	0 (0,0)	3 (7,5)	3 (3,8)	0,015
3a	5 (12,5)	15 (37,5)	20 (25,0)	
3b	21 (52,5)	15 (37,5)	36 (45,0)	
4	12 (17,5)	7 (17,5)	19 (23,8)	
5	2 (5,0)	0 (0,0)	2 (2,5)	
Estágio da DRC na consultaT3, n (%)^*^			
2	0 (0,0)	8 (20,0)	8 (10,1)	<0,001
3a	3 (7,7)	13 (37,5)	16 (20,3)	
3b	15 (38,5)	13 (42,5)	28 (35,4)	
4	17 (43,6)	6 (17,5)	23 (29,1)	
5	4 (10,3)	0 (0,0)	4 (5,1)	

Notas – *Estágios da DRC de acordo com a eTFGcr (mL/min/1,73 m^2^): 2 (60–89); 3a (45–59); 3b (30–44); 4 (15–29); 5 (< 15).

**Valor p calculado utilizando o teste qui-quadrado.

## Discussão

Neste estudo, a hiperuricemia assintomática foi associada a um declínio na função renal, indicando que níveis elevados de ácido úrico podem contribuir para danos renais. Essa associação foi inicialmente observada no grupo controle, no qual houve uma progressão desfavorável da DRC. Subsequentemente, o benefício clínico da terapia redutora de urato foi demonstrado no grupo que recebeu alopurinol. Ao reduzir os níveis de ácido úrico, o tratamento com alopurinol levou a uma melhora na função renal. Isso sugere que a diminuição dos níveis de ácido úrico pode desempenhar um papel na preservação ou na melhora da função renal.

No grupo controle, observou-se que o protocolo de tratamento padrão para hiperuricemia assintomática não foi eficaz no controle dos níveis de ácido úrico, com o ácido úrico sérico aumentando constantemente ao longo das consultas de acompanhamento. Concomitantemente, a média da eTFGcr declinou progressivamente, levando à reclassificação dos pacientes em estágios de piora da doença renal. Notavelmente, dentro de 24 meses, quatro mulheres do grupo controle experimentaram um declínio na função renal a ponto de necessitar de terapia de substituição renal (RRT) baseada em diálise. A persistência de níveis elevados de ácido úrico tem sido associada a um risco aumentado de progressão para doença renal em estágio terminal^16^. Além disso, pesquisas destacaram uma correlação entre hiperuricemia e sexo, indicando uma maior probabilidade de mulheres experimentarem redução da eTFGcr devido ao depósito de urato. Nesse contexto, a hiperuricemia emerge como um potencial preditor independente de doença renal em estágio terminal em mulheres^17^.

Entre os 40 pacientes que receberam TRU com alopurinol, foi observada uma redução notável nos níveis médios de ácido úrico sérico ao longo de 24 meses, confirmando a eficácia do alopurinol na redução do ácido úrico sérico entre indivíduos com DRC e níveis de TFGe variando de 60 a 15 mL/min/1,73 m^2^. Além disso, vários estudos demonstraram consistentemente os benefícios sustentados e de longo prazo do alopurinol na redução dos níveis de ácido úrico e na concessão de vantagens para pacientes com DRC1^18^.

Em pacientes que receberam terapia contínua com alopurinol, houve um aumento consistente da média da TFGe em cada visita de acompanhamento, levando à reclassificação dos pacientes em estágios mais leves da doença renal. Destaca-se que nenhum dos indivíduos neste grupo progrediu para o estágio que exigia diálise ao longo do período de 24 meses, destacando as vantagens clínicas sustentadas da TRU com alopurinol para os desfechos renais nessa população específica.

Numerosos estudos têm demonstrado consistentemente melhora da função renal em pacientes submetidos à TRU com alopurinol, tanto em grupos de tratamento quanto em comparação com grupos controle^1,16,19^. Golmohammadi et al.^1^, por exemplo, revelaram descobertas convincentes que demonstram redução substancial nos níveis de ácido úrico sérico, diminuição dos níveis de creatinina sérica e melhora da TFGe entre indivíduos com DRC leve após 12 meses de uso de alopurinol. Da mesma forma, Goicoechea et al.^16^ relataram melhora na taxa de filtração glomerular estimada após 24 meses de terapia com alopurinol. Em um estudo subsequente, publicado em 2015, os mesmos autores demonstraram resultados consistentes com o tratamento com alopurinol por até 84 meses, concluindo que a terapia prolongada pode mitigar a taxa de progressão^20^. Além disso, uma revisão sistemática de ensaios clínicos randomizados, realizada em 2017, sugeriu que os inibidores da xantina oxidase podem oferecer benefícios diretos aos rins além de seus efeitos hipouricêmicos. Nessa revisão, ensaios que utilizaram alopurinol exibiram desaceleração na queda da TFGe ao longo do tempo e redução no risco de progressão para doença renal em estágio terminal^19^.

Os níveis elevados de urato sérico e ácido úrico urinário têm sido implicados em eventos renais adversos e podem contribuir para a progressão da DRC, embora o mecanismo preciso subjacente ao dano renal permaneça elusivo. Investigações patofisiológicas em modelos animais revelaram que a hiperuricemia desencadeia processos inflamatórios que promovem respostas vasoativas, retenção de sódio, constrição vascular e aumento da pressão arterial^21^. Evidências experimentais sugerem que o ácido úrico atua como um potente ativador do SRAA em humanos^22^, além de ativar outros vasoconstritores cruciais, como a endotelina e o tromboxano, enquanto inibe simultaneamente as vias vasodilatadoras, como o óxido nítrico^23^. Além disso, a elevação do ácido úrico promove estresse oxidativo intracelular, levando à inflamação, proliferação celular e fibrose renal^4^. Ademais, a uricosúria e a presença de cristais de urato podem induzir danos tubulares por meio de mecanismos diretos ou instigando inflamação tubulointersticial^24^.

O impacto da TRU com alopurinol na progressão da DRC permanece uma área de pesquisa em andamento. Estudos experimentais realizados em ratos demonstraram que o alopurinol previne efetivamente o início da hipertensão e modula os níveis de renina e óxido nítrico, mitigando, assim, os efeitos hipertensivos associados à hiperuricemia^21^. Em um estudo de Yelken et al.^25^, foi demonstrado que o tratamento com alopurinol para hiperuricemia reduziu o estresse oxidativo, melhorou a disfunção endotelial e a função renal em pacientes com DRC. Outro estudo realizado por Bayram et al.^12^ explorou a acidose metabólica como um fator de risco para a progressão da DRC e demonstrou que a administração de alopurinol levou à diminuição nos níveis de ácido úrico e ao aumento do bicarbonato sérico, o que poderia ajudar a prevenir a acidemia e retardar a progressão da doença renal.

Ao inibir a xantina oxidase, uma enzima responsável por produzir superóxido, o alopurinol exerce um efeito antioxidante crucial, controlando a geração de radicais livres de oxigênio e desativando vias pró-inflamatórias implicadas na progressão da DRC^26^. Esses achados, em conjunto, sugerem que o alopurinol pode ter potencial como intervenção terapêutica na mitigação da progressão da DRC, embora mais pesquisas sejam necessárias para elucidar seus mecanismos precisos e sua eficácia terapêutica.

Todos os 80 pacientes em nosso estudo receberam o tratamento padrão para DRC e hiperuricemia assintomática. Consequentemente, inferimos que a melhora na função renal observada no grupo que recebeu alopurinol decorreu da redução do ácido úrico por meio da TRU. Ao longo de nossa pesquisa, a administração de alopurinol, com dose média próxima a 200 mg por dia, reduziu efetivamente os níveis de ácido úrico para abaixo de 5 mg/dL ao final do período de acompanhamento. Esse valor médio de ácido úrico, de aproximadamente 5 mg/dL, pode indicar um nível alvo potencialmente benéfico para a proteção da função renal^27^ ou sugerir valores de corte para definir hiperuricemia assintomática inferiores aos tradicionalmente utilizados^28^.

Avaliar o impacto da hiperuricemia em indivíduos com DRC representa um desafio considerável devido à sua complexidade. A associação negativa observada entre a hiperuricemia assintomática e a DRC pode ter sido influenciada por vários outros fatores de risco presentes em nossa população, incluindo idade, peso, dieta, hipertensão, diabetes, medicamentos que afetam a excreção de ácido úrico e proteinúria. Notavelmente, a idade média dos participantes ultrapassou 60 anos, sem variações discerníveis entre os grupos caso e controle. Considerando que a função renal diminui com a idade devido à perda progressiva de néfrons, o envelhecimento populacional emerge como um fator de risco significativo para DRC^28^. No entanto, pesquisas indicam que níveis elevados de ácido úrico sérico em populações idosas e de meiaidade correlacionam-se negativamente com a TFGe^29^.

Os pacientes no grupo alopurinol apresentaram pesos corporais significativamente mais altos em comparação aos do grupo controle, alinhando-se potencialmente a descobertas de outros estudos que sugerem uma associação direta entre sobrepeso e obesidade e níveis de ácido úrico sérico^30,31^. Entre todos os pacientes em nosso estudo, 52,5% tinham hipertensão e 22% tinham diabetes como causa primária da DRC, ambos reconhecidos como fatores de risco para danos renais progressivos e potenciais variáveis de confusão em nossa população. No entanto, vários estudos estabeleceram uma ligação direta entre níveis elevados de ácido úrico e um aumento do risco de hipertensão e diabetes^4,21^.

Recentemente, dois estudos avaliaram a eficácia do alopurinol em interromper a progressão da DRC, mas nenhum deles demonstrou benefícios clínicos significativos para os pacientes que usaram o medicamento^32,33^. Esses estudos reconheceram que alguns participantes recrutados não apresentaram hiperuricemia no início; assim, uma parte dos sujeitos tinha níveis normais de ácido úrico sérico. Em contraste, nosso estudo observacional retrospectivo incluiu exclusivamente pacientes com hiperuricemia assintomática, garantindo consistência ao longo do período de acompanhamento de 24 meses, sem perdas de seguimento.

Outro estudo avaliou os mecanismos biológicos desencadeados pela elevação do ácido úrico solúvel e do ácido úrico cristalino separadamente, discutindo o papel da hiperuricemia assintomática na DRC, na CVD e na inflamação estéril, apontando para possibilidades de uso da terapia redutora de ácido úrico em pacientes com doença renal, especialmente quando cristais de ácido úrico são identificados na urina^34^. Embora muitas diretrizes atuais, como a KDIGO 2024^35^, não recomendem o tratamento rotineiro da hiperuricemia assintomática para mitigar a progressão da DRC devido à limitação das evidências disponíveis, o debate persiste há anos, apoiado por modelos experimentais conflitantes e dados clínicos sugerindo um papel patogênico do ácido úrico no declínio indesejável da função renal. Estudos recentes questionaram o potencial benefício de tratar a hiperuricemia assintomática na desaceleração da progressão da DRC. O ensaio clínico randomizado FIX-CKD, um dos mais robustos realizados até agora, não mostrou efeito do alopurinol na atenuação da queda da função renal^33^. Além disso, meta-análises publicadas nos últimos anos relataram resultados inconsistentes e heterogeneidade substancial entre os estudos, relacionados a diferenças nos critérios de inclusão, características populacionais, controle de comorbidades e duração do acompanhamento. Essa variabilidade metodológica dificulta a capacidade de tirar conclusões definitivas e reforça a necessidade de ensaios controlados randomizados bem planejados, com variáveis clínicas padronizadas e acompanhamento de longo prazo^36,37^.

Uma limitação em nosso estudo foi a inclusão de medicamentos que modificam a excreção de ácido úrico, em especial diuréticos, ácido acetilsalicílico em baixa dose, certos anti-hipertensivos e medicamentos redutores de lipídios^38,39^. Embora esse efeito não tenha sido estatisticamente significativo, a utilização desses medicamentos pode ter influenciado os níveis de ácido úrico sérico, e esse aspecto não foi examinado isoladamente. Além disso, é importante enfatizar que não conseguimos obter o índice de massa corporal (IMC) dos pacientes devido à ausência de instrumentos apropriados para a medição da altura na clínica ambulatorial. Ressalta-se que os pacientes receberam atendimento por meio do sistema público de saúde, o qual é o sistema predominante no país. A importância da proteinúria como fator de risco para a progressão da DRC é amplamente reconhecida^29^. No entanto, em nosso estudo, os pacientes avaliados não utilizaram medicamentos antiproteinúricos das classes de inibidores de SGLT2 e nsMRA. Dessa forma, não analisamos a influência da proteinúria na DRC, o que poderia potencialmente introduzir viés. Vale ressaltar que, embora a proteinúria não tenha sido estatisticamente significativa ao comparar ambos os grupos, sua omissão em nossa análise deve ser considerada. Além disso, a duração do acompanhamento dos pacientes e o tamanho da amostra também podem ter limitado os resultados apresentados.

Outra limitação importante deste estudo diz respeito à potencial influência de comorbidades como hipertensão, diabetes e excesso de peso corporal, que são altamente prevalentes entre indivíduos com DRC e podem atuar como fatores de confusão na relação entre hiperuricemia e função renal. Essas condições apresentam mecanismos patofisiológicos que afetam tanto os níveis de ácido úrico sérico quanto a taxa de filtração glomerular, dificultando a determinação de um efeito causal direto. Embora tenhamos descrito essas variáveis e comparado sua distribuição entre os grupos, a ausência de controle sistemático ou de ajuste estatístico para esses fatores impede conclusões definitivas sobre a independência da associação observada. Portanto, nossas descobertas devem ser interpretadas com cautela, sublinhando a necessidade de estudos prospectivos com coleta padronizada de comorbidades e de potenciais variáveis de confusão, a fim de elucidar de forma mais robusta o papel da hiperuricemia assintomática na progressão da DRC^27,40^.

Concordamos que ensaios clínicos randomizados adicionais, controlados por placebo, são imperativos para tirar conclusões definitivas sobre o aumento do risco de progressão da DRC associado à hiperuricemia assintomática, bem como sobre a eficácia e a segurança da utilização do alopurinol para reduzir os níveis de ácido úrico sérico e mitigar a queda na TFGe em vários estágios da DRC. Por fim, vale destacar que a população do estudo, baseada no Brasil, consiste em pacientes tratados no Sistema Único de Saúde (SUS), um sistema público de saúde financiado pelo Estado e acessível à população de baixa renda. É importante enfatizar que o tratamento com alopurinol constitui uma ferramenta relevante disponibilizada gratuitamente aos pacientes com DRC e hiperuricemia, sublinhando a importância dessa estratégia farmacológica.

## Conclusão

Neste estudo, determinamos que a TRU baseada em alopurinol constitui uma abordagem valiosa na redução dos níveis de ácido úrico sérico e na melhora da função renal entre pacientes com doença renal crônica apresentando hiperuricemia assintomática, durante um período de acompanhamento ambulatorial de 24 meses. As doses administradas de alopurinol foram seguras e eficazes, resultando consistentemente em aumento da TFGe em cada visita de revisão e ajudando a retardar a progressão para a insuficiência renal. Nossas descobertas reforçam a importância de considerar a TRU com alopurinol para pacientes ambulatoriais com doença renal crônica (DRC) e hiperuricemia assintomática, seguindo orientações adequadas e acompanhamento por meio de consultas regulares durante todo o período.

## Data Availability

Os conjuntos de dados gerados e/ou analisados durante o presente estudo estão disponíveis mediante solicitação razoável ao autor correspondente.
